# Work-Related Stress, Health Status, and Status of Health Apps Use in Korean Adult Workers

**DOI:** 10.3390/ijerph19063197

**Published:** 2022-03-08

**Authors:** Won Ju Hwang, Minjeong Kim

**Affiliations:** 1 College of Nursing Science, East-West Nursing Research Institute, Kyung Hee University, Seoul 02447, Korea; hwangwj@khu.ac.kr; 2 School of Nursing, San Diego State University, San Diego, CA 92182, USA

**Keywords:** Koreans, mobile applications, occupational stress

## Abstract

Although health apps have been developed and utilized in many countries, there is no baseline study about what percentage of Korean workers use these types of health apps. Therefore, the purpose of this study was to describe the work-related stress, health status, and utilization of health apps of Korean adult workers. This descriptive study included 95 adults in South Korea. Demographic variables, work-related stress, health status, and utilization of health apps were obtained using an online self-reported survey. Descriptive analyses were used to explore prevalence of each variable. This study found that almost 65% of the participants fell into the higher work-related stress group. About 41.6% of the participants in this sample evaluated their general health status as fair to poor with 26.8% being overweight to obese and 11.6% having hypertension. However, only about 33.7% of the sample have used health apps. Therefore, utilization of health apps as health and stress management tools should be encouraged at a public health level.

## 1. Introduction

Mental health promotion in adults has great public health concerns and social significance [1]. According to the results of the Korean Working Conditions Survey (KWCS), the number of workers who experienced depression and anxiety in the past 12 months was 1.6% in 2010, 1.5% in 2011, and 1.4% in 2014. Considering the reluctance to expose mental symptoms due to stigma at work and difficulties in returning to work after treatment, it is expected that the number of workers will continue to increase in the future [2].

As of 2021, the number of Korean workers was 18,945 thousand, which is 36.7% of the total population in Korea. According to classification of industries, the number of workers in manufacture was the largest (*n* = 3675 thousands), followed by sales (*n* = 2286 thousands), health and social work service (*n* = 2045 thousands), education service (*n* = 1677 thousands), and construction (*n* = 1378 thousands). Korean workers reported that they worked 149 h per month and their monthly income was around $3075 [3].

### 1.1. Work-Realted Stress and Health Status of Korean Adult Workers

Recent studies of Korean workers’ stress found that Korean workers have experienced high levels of stress at work [2,3,4,5]. Park, Kook [2] found that over-time working significantly increased stress levels in Korean young adult workers. Job insecurity, a lack of reward, and uncomfortable organizational environment have also been reported as work-related stressors in Korean workers, which are typical Asian work environment which place a heavy emphasis on diligence and senior authority [6,7]. More specifically, studies of health care workers’ stress found that professional burnout was related to higher work-related stress in medical doctors [8] and high emotional labor was one of the stressors in interpersonal service workers in hospitals [9].

Relationships between work-related stress and health problems have already established in numerous studies. Some studies found that over-time working, job insecurity, a lack of reward, and uncomfortable organizational environment significantly increased the risk of depression, suicidal ideation, and musculoskeletal disease in Korean workers [2,6,7,10]. In regards to specific types of job, work-related stress significantly increased depression, anxiety, and sleep problems in Korean dentists [11] and suicidal ideation in Korean firefighters [12]. Emotional labor and job insecurity also significantly increased the risk of depression in female call center workers [13].

Similar relationships between work-related stress and health problems have also been found in studies with Asian and various populations. Higher levels of over-commitment and less organizational support significantly increased the risk of chronic fatigue syndrome in Chinese nurses [14]. Higher stress significantly increased the risk of hypertension in 530 Asian Americans [15], and alterations in circadian rhythm increased systolic and diastolic blood pressure in night shift workers in airline companies [16]. Two studies of diabetes found that work-related stress significantly increased blood glucose level (HbA1C) [17] and the incidence of both prediabetes and diabetes [18].

Although Korean adult workers experience work-related stress and even stress-related health problems, very few of them have utilized professional mental health service [19,20], which might be due to a lack of knowledge on self-care including stress management and not enough available resources in Korea. Since the onset of the COVID-19 pandemic, public health orders including stay-at-home and physical distancing measures have put people in more stressful situations, such as financial difficulties and changes from in-person to online work environments. It is also evident that people have been reluctant to see a doctor for the management of their current health conditions due to the fear of getting infected with COVID-19 in health care settings, resulting in undermanaged health problems [21].

### 1.2. Status of Health Apps Use of Korean Adult Workers

Very recently, various health apps have been developed and utilized to help track their blood pressure, blood sugar, food, exercise, weight, and even medications for prevention and self-care purposes in Korea as well as other countries. Examples of health apps that are widely used in Korea include “WishCare” [22], “MyTherapy” [23], and “HeLpy” [24]. In addition to physical health apps, stress care apps have also been developed to manage their stress and anxiety, featuring recommendations for mindfulness-based breathing techniques, meditation, and healing sounds based on their stress and anxiety level (in mind [25], KLAR [26], and “MindHealer” [27]).

Recently, studies of effects and efficacy of the health apps have been conducted in other countries. Specifically, mindfulness meditation apps significantly decreased perceived stress level in 88 medical students in the Unites States (Happy Healers) [28] and in 74 adults in the United Kingdom (Headspace) [29], and significantly decreased job strain, distress, and systolic blood pressure in 238 healthy employees in the United Kingdom [30]. Music-based emotion regulation apps (Music eScape) was also effective in improvement of emotional regulation skills and well-being in 169 young people with mild mental distress [31]. A study of effectiveness of an integrated health apps which offers diet, physical activity, sleeping habits, stress, and alcohol use, is ongoing in 209 Sweden adult workers [32]. Although many clinical trials about the effectiveness of health apps have been emerged in many countries and found their effectiveness on health, there is no baseline study about what percentage of Korean workers use these types of health apps. Therefore, this baseline descriptive study looking at work-related stress and health status and status of health apps use in Korean workers, is needed first.

### 1.3. Purpose of the Present Study

Therefore, the purpose of the present study was (1) to describe work-related stress and health status in a sample of Korean adult workers and (2) describe status of health apps use in this sample.

## 2. Methods

### 2.1. Study Design

This descriptive study used a cross-sectional design to describe work-related stress and health status, and status of health apps use in the Korean adult workers.

### 2.2. Participants and Setting

This web-based survey study included 95 adult workers in South Korea between November 2018 and December 2018. The inclusion criteria were: (1) self-identified as Korean; (2) between 18 and 65 years old; (3) able to communicate in Korean; (4) has an electronic device, such as a cellphone, tablet, or PC; (5) understands the purpose of this study. All participants who did not meet the inclusion criteria were excluded.

### 2.3. Procedure

Potential participants were reached out using social media in Seoul, South Korea. A convenience sampling method was employed. A 4-part, web-based self-report survey was developed using the Survey Monkey software and a survey link was sent to potential participants. Potential participants were also asked to share this study information with their friends if they are eligible for this study. A potential participant proceeded with this study if s/he was interested.

### 2.4. Measures

#### 2.4.1. Demographic Questionnaire

A 6-item investigator-developed demographic questionnaire was used. The items included questions on gender (male and female), age (in years), educational level (high school or below, college or Bachelor degree, and above graduate school), marriage status (single, married, and others), types of job (white-collar, blue-collar, and others), and monthly family income ($1809.00 or below, $1809.00–$3617.00, and above $3617.00).

#### 2.4.2. Work-Related Stress

A short version of the Effort–Reward Imbalance Questionnaire (ERIQ) is a self-report, 4-point Likert scale to measure participants’ effort, reward, and over commitment at work: Effort Scale (ES, 3 items), Reward Scale (RS, 7 items), and Over Commitment (OC, 4 items). A sum of each subscale ranges from 3–12 for the ES, 7–28 for the RS, and 4–16 for the OC. The Effort/Reward(ER) ratio was computed by placing the effort score in the enumerator and the reward score in the denominator. The latter score was multiplied by a correction factor prior to being placed in the denominator to adjust for the unequal number of items. Correction factor was 0.4286 when the enumerator contained 3 items and the denominator contained 7 items. An ER ratio higher than 1.0 indicates an imbalance between high effort and low reward [33]. Cronbach alphas for our sample of Korean adults were 0.68 for the ES, 0.80 for the RS, and 0.83 for the OC.

#### 2.4.3. Health Status

Participants’ health status was assessed by three measures: general health status, Body Mass Index (BMI), and health problems. To assess the participants’ evaluation of their overall health, one question was answered on a five-point scale consisting of the answers excellent, very good, good, fair, and poor. BMI was computed by putting weight in kilograms in the enumerator and height in meters squared in the denominator. Scores below 18.5 are considered underweight, scores between 18.5 and 24.99 for normal, scores between 25.0 and 29.99 for overweight, and scores above 30.0 for obese [34]. Health problems were noted as yes/no for hypertension, hyperlipidemia, and diabetes.

#### 2.4.4. Status of Health Apps Use

A 1-item investigator-developed questionnaire was used. The item asked if what types of health apps has been ever used at work or at home: overall health, mental health-focused, or physical health-focused apps.

### 2.5. Data Analysis

SPSS 26.0 version (IBM, Armonk, NY, US)program was used to analyze the data in this study. Descriptive statistics were used to examine demographic characteristics, including frequency and percent for gender, educational level, marriage status, types of job, and monthly family income, and mean (*M*) and standard deviations (SD) for age. *M* and SD were also calculated for the work-related stress, and frequency and percent for general health status, BMI, health problems, and types of health apps use.

## 3. Results

Participants consisted of 32 males (38.1%) and 52 females (61.9%). The mean age of the participants was 40.3 years (32–47 years, SD = 7.4). About 92.9% had college or higher education levels. About 71.4% of the participants were married. About 79.1% of the participants reported that they were white-collar workers. About 57.8% of the participants reported that “their family earns higher than $3617.00 monthly” and 38.6% reported that “their family earns between $1809.00 and $3617.00 monthly” (Table 1).

Participants’ levels of work-related stress are presented in Table 2. The mean score of the ES was 9.0 (SD = 1.9) and 19.1 (SD = 4.4) for the RS. The ER ratio was 1.2 (SD = 0.6), which indicated that about 64.8% of the participants fell into a higher work-related stress group and the other 35.2% of the participants fell into a lower work-related stress group. The mean score of the OC was 10.0 (SD = 3.3).

As shown in Table 3, about 41.6% of the participants fell into either the “fair” or “poor” health status groups, whereas only 19.1% of the participants fell into either the “excellent” or “very good” health status groups. About 26.8% of the participants were in either the “overweight” or “obese” group. Among health problems, the prevalence of hypertension was the greatest (*n* = 11, 11.6%), followed by hyperlipidemia (*n* = 6, 6.3%) and diabetes (*n* = 4, 4.2%).

Participants’ status of health apps use are presented in Table 4. About 33.7% of the participants reported that they have ever used health apps. More specifically, about 10.1% (*n* = 9) of the participants have ever used mental health-focused apps, and only 4.5% (*n* = 4) for physical health-focused apps.

## 4. Discussion

This study explored Korean adult worker’s work-related stress, health status, and status of health apps use. Of demographic variables, most of our study participants (79.1%) fell into a white-collar employee group. Moreover, about half of our study participants had a graduate school or higher educational level (43.5%) and earned $3617.00 or higher per month (57.8%), which may be due to a selection bias of a convenience sampling. Therefore, repeated studies with more representative samples of Korean adult workers including those with low socio-economic status or vulnerable population, are needed.

Higher work-related stress was reported by 64.8% of the participants in this study, which was similar or quite higher than those from other studies that reported Korean workers reported higher work-related stress [2,3,4,5]. This finding indicated that Korean workers have put a lot of effort, energy, and commitment towards their accomplishments at work, but have received quite less rewards than they deserve, considering the negative adverse work environments typically seen in Asian culture [6,7].

The study findings about the prevalence of hypertension (11.6%) was inconsistent with those from other reports which showed 3.3% of hypertension rate among 4865 employed pregnant women from the Amsterdam Born Children and their Development data [35], 5.5% in Chinese patients [36], 19.4% in Chinese petroleum workers [37], and 33.3% in Egyptian bus drivers [38]. The prevalence of 4.2% of diabetes rate in this study was comparable to those from other studies which found 2.2% of diabetes rate from the Brazilian Longitudinal Study of Adult Health [18] and 3.4% of cardio-metabolic disease rate (coronary heart disease, stroke, or diabetes) from the European Work Consortium data [39]. Although there were some inconsistent findings regarding participants’ health status, our understanding is that our participants’ sedentary lifestyle might contribute to increased hypertension and diabetes rate [2,6,7,40,41]. This study did not look at the relationships among types of jobs, work-related stress, and health status, therefore, further analyses are needed.

Our participants in this study reported use of overall health apps (33.7%), mental health-focused apps (10.1%), and physical health-focused apps (4.5%). Study findings about the utilization of health apps were hard to compare with those from other studies because of a lack of studies on the prevalence of health apps use in the adult worker populations [28,29,30,31,32]. This underutilization of health apps might be due to a lack of health apps available in the Korea, a lack of research study about the effectiveness of health apps as health and stress management tools, and a lack of public awareness about the importance of health apps use in improving their health status. Further analyses are also needed to identify factors that may contribute to the lower use of health apps. Since the COVID-19 pandemic, the need for self-care and stress managements has significantly increased. Therefore, the utilization of health apps should be encouraged at public health level.

Limitations of this study should be noted. Due to a convenience sampling method, our study findings may not be generalized to all Korean adult workers. This study used a self-report online survey for work-related stress and health status that might be often under-reported due to stigma around stress and health issues. Moreover, this study did not analyze the relationships among work-related stress, health status, and health apps use so causal relationship cannot be guaranteed. Despite the study limitations, this study is significant in that this study was the very first study that addressed work-related stress and health apps use in Korean adult workers.

## 5. Conclusions

This study found that Korean adult workers experienced higher levels of work-related stress, fair to poor health status, overweight, and hypertension. Participants underutilized health apps. Health apps use which help tracking and managing their stress and health should be encouraged for Korean adult workers.

## Figures and Tables

**Table 1:** Demographic characteristics of Korean workers (*n* = 95, valid case only).

Variable	*n* (%)/M (SD)
Gender	
Male	32 (38.1)
Female	52 (61.9)
Age	40.3 (7.4)
Educational level	
High school or below	6 (7.1)
College or Bachelor degree	42 (49.4)
Graduate school or higher	37 (43.5)
Marriage status	
Single	21 (25.0)
Married	60 (71.4)
Others (divorce, etc.)	3 (3.6)
Types of job	
White-collar	68 (79.1)
Blue-collar	4 (4.7)
Others	7 (8.1)
Unemployed	7 (8.1)
Monthly family income	
$1809.00 or below	3 (3.6)
$1809.00–$3617.00	32 (38.6)
$3617.00 or higher	48 (57.8)

**Table 2:** Levels of work-related stress of Korean workers (*n* = 95, valid case only).

Variable	M (SD)/*n* (%)
Effort-Reward Imbalance Questionnaire (ERIQ)	
Effort Scale (ES)	9.0 (1.9)
Reward Scale (RS)	19.1 (4.4)
Effort-Reward (ER) ratio	1.2 (0.6)
≥1.0 (higher stress group)	57 (64.8)
<1.0 (lower stress group)	31 (35.2)
Over Commitment (OC)	10.0 (3.3)

**Table 3:** Health status of Korean workers (*n* = 95).

Variable	*n* (%)
General health status	
Excellent	3 (3.4)
Very good	14 (15.7)
Good	35 (39.3)
Fair	29 (32.6)
Poor	8 (9.0)
Body Mass Index (BMI)	
18.5 or below (underweight)	5 (5.8)
18.5–24.9 (normal)	58 (67.4)
25.0–29.9 (overweight)	22 (25.6)
30.0 or higher (obese)	1 (1.2)
Health problems (multiple response)	
Hypertension	11 (11.6)
Hyperlipidemia	6 (6.3)
Diabetes	4 (4.2)

**Table 4:** Status of health Apps use of Korean workers (*n* = 95).

Variable	*n (%)*
Types of health apps use (multiple response)	
Overall health	30 (33.7)
Mental health-focused	9 (10.1)
Physical health-focused	4 (4.5)

## Data Availability

Please contact the corresponding author for data availability.
